# The Use of and Experiences With Telelactation Among Rural Breastfeeding Mothers: Secondary Analysis of a Randomized Controlled Trial

**DOI:** 10.2196/13967

**Published:** 2019-09-03

**Authors:** Kandice Kapinos, Virginia Kotzias, Debra Bogen, Kristin Ray, Jill Demirci, Mary Ann Rigas, Lori Uscher-Pines

**Affiliations:** 1 RAND Corporation Arlington, VA United States; 2 University of Pittsburgh School of Medicine Pittsburgh, PA United States; 3 University of Pittsburgh School of Nursing Pittsburgh, PA United States; 4 UPMC Cole Hospital Coudersport, PA United States

**Keywords:** telehealth, telemedicine, breastfeeding, lactation, lactation support, rural health

## Abstract

**Background:**

Telelactation services connect breastfeeding mothers to remotely located lactation consultants through audio-visual technology and can increase access to professional breastfeeding support in rural areas.

**Objective:**

The objective of this study was to identify maternal characteristics associated with the demand for and use of telelactation and to describe visit characteristics.

**Methods:**

We conducted a descriptive study within the context of a randomized controlled trial. Participant survey data and vendor electronic medical record data were used to assess video call characteristics like timing, duration, topics discussed, and participant satisfaction. Recruitment occurred from 2016-2018 at a rural critical access hospital in Pennsylvania. The 102 women enrolled in the study were given access to unlimited, on-demand video calls with lactation consultants through a mobile phone app and were tracked for 12 weeks following their postpartum hospitalization.

**Results:**

A total of 94 participants out of 102 recruits (92%) participated in the final, 12-week survey assessment were included in the analysis. Of those, 47 (50%) participants reported participating in one or more video calls, and 31 (33%) completed one or more calls that included a substantive discussion of a breastfeeding challenge. Participants who used telelactation (21/31, 68%; *P*=.02) were more likely to be working at 12 weeks postpartum compared to others (26/63, 41%), were less likely (12/31, 39%; *P*=.02) to have prior breastfeeding experience on average compared to nonusers (41/63, 65%), and were less likely to have breastfed exclusively (16/31, 52%; *P*<.001) prior to hospital discharge compared to mothers who didn’t use telelactation services (51/63, 81%). Most video calls (58/83, 70%) occurred during the infant’s first month of life and 41% (34/83) occurred outside of business hours. The most common challenges discussed included: breast pain, soreness, and infection (25/83, 30%), use of nipple shields (21/83, 25%), and latch or positioning (17/83, 24%). Most telelactation users (43/47, 91%) expressed satisfaction with the help received.

**Conclusions:**

Telelactation is an innovation in the delivery of professional breastfeeding support. This research documents both demand for and positive experiences with telelactation in an underserved population.

**Trial Registration:**

ClinicalTrials.gov NCT02870413; https://clinicaltrials.gov/ct2/show/NCT02870413

## Introduction

Breastfeeding has multiple benefits for mothers, infants, and society, helping to protect against a variety of short- and long-term health conditions and reducing associated healthcare costs [1-4]. Unfortunately, the majority of women in the United States stop breastfeeding before it is recommended, with only 36% of infants receiving any breastmilk at 12 months of age [5]. There are also significant disparities in breastfeeding rates [6], with rural and low-income mothers less likely to initiate and maintain breastfeeding, thus putting them at greater risk for a range of negative health outcomes [7,8].

One strategy to improve breastfeeding rates, particularly among underserved populations, is to increase access to professional breastfeeding support by International Board-Certified Lactation Consultants (IBCLCs). In fact, the Surgeon General’s Call to Action to Support Breastfeeding identifies increasing access to IBCLCs as a policy priority [9] because research has shown that they improve breastfeeding duration and exclusivity (ie, proportion of breastmilk in an infant’s diet) [10-13]. Although a range of professionals provide breastfeeding support and address breastfeeding complications, IBCLCs are exclusively focused on breastfeeding in contrast to other medical professionals who must balance a range of competing demands during each visit [14,15]. In rural communities, poor access to IBCLCS may contribute to breastfeeding disparities [16-20].

Telelactation is one tool that can be leveraged to increase access to IBCLCs in rural settings [21]. Telelactation services connect breastfeeding mothers to remotely located IBCLCs through audio-visual technology. Several companies, including Medela, Lansinoh, American Well, and Pacify Health, currently offer telelactation video visits to breastfeeding women through their mobile apps [21,22], but the current published literature on this particular form of mobile telelactation is limited to one qualitative study on mothers’ experiences [22]. Furthermore, there is no research on who uses telelactation, or the content, duration, and timing of these visits.

In the context of a pilot randomized controlled trial on telelactation in a rural population, we set out to address this gap in the literature. We aimed to use survey and electronic medical record (EMR) call log data to identify maternal characteristics associated with demand for and use of telelactation, to describe telelactation visit characteristics, and to describe experiences with visits.

## Methods

### Overview

From October 2016 to May 2018, we recruited rural women into a randomized controlled trial of telelactation. Participants were enrolled during their postpartum hospitalization at a critical access hospital in North-Central Pennsylvania and tracked for 12 weeks following enrollment. The hospital is located in a Health Professional Shortage Area (HPSA) within a county designated as 100% rural by the 2010 United States Census. At the time of the study, it had approximately 200 births per year and did not have any IBCLCs on staff or practicing within 40 miles of the hospital. At the time recruitment began, breastfeeding initiation rates among the hospital’s patients (74%) were below the national average (80%) [23].

Women were eligible to participate if they were >18 years old, had an infant with a gestational age of > 35 weeks, had initiated breastfeeding and planned to continue after hospital discharge, and did not have a condition incompatible with breastfeeding (eg, HIV positive) [24]. Following informed consent, participants were enrolled into one of two study arms: telelactation (intervention) or usual care (control). Participants randomized to the telelactation arm were given an orientation to Pacify Health’s telelactation app by hospital nurses involved in study recruitment. The nurses showed participants how to download the app on their personal device, provided a coupon code for free, unlimited video visits, and helped participants conduct a test call. After this orientation, participants could request unlimited, on demand video calls with IBCLCs through the app. The telelactation app used in the trial was designed for video calls to occur within seconds of visit initiation by the mother. In addition, it is Health Insurance Portability and Accountability Act (HIPAA)–compliant and involves a large network of geographically dispersed IBCLCs available to take video calls 24 hours a day. Participants randomized to the control arm received care as usual (ie, no additional lactation support beyond what is typically received in the hospital and in outpatient settings). These participants were not included in this analysis because our aim was to describe use of, and experiences with, telelactation among those given access to it. Control arm participants did not have access to these services.

### Data and Sample

We used two data sources to assess use of and experiences with telelactation: participant survey data and EMR call log data supplied by the telelactation vendor.

### Survey Data

We fielded online surveys to all study participants at baseline (enrollment in the hospital) and at 2, 4, and 12 weeks postpartum. All participants received questions on demographics (baseline survey), breastfeeding practices (all surveys), breastfeeding plans (baseline survey), work plans and status (baseline and 12-week surveys), and breastfeeding challenges (2-week and 12-week surveys). However, participants enrolled in the telelactation arm (n=102) received an additional module on experiences with the telelactation app at 2, 4, and 12 weeks. This module included questions on their use of the telelactation app, challenges with using the app, satisfaction with the app, and recommendations for improvement. For this study, we restricted our sample to telelactation arm participants who completed the final 12-week survey (n=94), which represents 92% of the subjects in this arm.

### Electronic Medical Record Data

The telelactation vendor provided a log of all the video calls completed by study participants during the study period. For each video call with clinical content (n=83), the vendor supplied patient identification, call duration, date and time of call, and reason for call or issues discussed. We merged the individual-level survey data with the EMR call log data to examine how telelactation arm participants who used the app differed from those who did not.

### Measures

We applied two definitions of telelactation use. First, we defined telelactation use based on vendor EMR data. Any participant with one or more documented video calls that addressed a breastfeeding challenge was classified as a telelactation user for the analyses comparing users to nonusers and characterizing visits. Second, for analyses on satisfaction and experiences with the app, we relied on participants’ self-reported telelactation use. For this measure, participants who reported participating in one or more video calls during the study period in the 12-week survey, including for test or orientation calls that did not specifically address a breastfeeding issue, were classified as telelactation users. We applied the definition based on EMR data for most analyses because it is likely more accurate (ie, not subject to recall bias and social desirability bias) and captures meaningful use of the app. However, we also applied the definition based on participants’ self-reported use in some analyses to represent the perspectives of all participants who engaged with the app in any manner. Furthermore, it is possible that select participants accessed IBCLC support on the app in a way that was not recorded by the vendor (eg, through an account of a healthcare provider involved in the study), and we wanted to capture the experiences of those participants.

We included two measures of participants’ overall assessment of telelactation as reported in the final 12-week survey. First, participants were asked to rate, “How helpful was the help you received through the app?” (very helpful, helpful, neither helpful nor unhelpful, somewhat helpful, or not at all helpful). Second, they were asked to indicate, “How satisfied were you with the help you received through the app?” (very satisfied, satisfied, neither satisfied nor unsatisfied, somewhat satisfied, and not at all satisfied). For both measures, we collapsed the two positive categories (ie, very satisfied and satisfied) and three neutral or negative categories (eg, neither satisfied nor unsatisfied, somewhat satisfied, and not at all satisfied) to create binary variables: helpful (yes or no) and satisfied (yes or no). Finally, using vendor data on video call date and time, we constructed a measure of visit timing. All calls that occurred between 6:01 PM-7:59 AM were defined as occurring outside of regular business hours.

### Statistical Approach

First, we examined whether participants who used telelactation differed from those who did not on multiple, self-reported, sociodemographic characteristics by using Chi-square tests and *t* tests for categorical and continuous variables, respectively. Second, we assessed telelactation visit characteristics. We calculated sample statistics and two-tailed *P* values to determine whether means across groups were statistically different using a *P* value of <.05. This study was reviewed and approved by the RAND Corporation’s Institutional Review Board (IRB). The recruitment hospital deferred to RAND’s IRB.

## Results

### Participants’ Use of Telelactation

Of the 102 women randomized to the telelactation arm within the trial, 94 (92%) completed the final, 12-week survey. Of these, 47 (50%) self-reported participating in one or more video calls during the study period. Using EMR data, we identified 31 (33%) participants who completed one or more calls that included a substantive discussion of a breastfeeding challenge. Among these 31 participants who discussed at least one substantive challenge through the app, 14 (45%) had one call only, 8 (26%) had 2 calls, and 9 (29%) had 3 or more calls.

Among the 94 participants, 68 (72%) reported experiencing one or more breastfeeding challenges during the study period. Of the 26 women who did not report breastfeeding challenges, none of them participated in a substantive discussion about a breastfeeding challenge via the app. As such, the rate of uptake for substantive video calls among women who reported challenges (ie, reported a potential need for lactation support) was 45% (31/68).

In Table 1, we present descriptive statistics on the full sample of participants in the telelactation arm that are also stratified by telelactation user versus nonuser. Overall, the mean age of the maternal participants was 26 years (SD 5) at baseline. In addition, 44 (47%) had a high school degree or less, 45 (48%) had private health insurance during pregnancy, 39 (41%) were first-time mothers and slightly more than half (49/94, 52%) planned to work for pay within 12 months of delivery.

**Table 1:** Characteristics of telelactation users versus nonusers (N=94).

Characteristics		Nonusers (n=63), n (%)	Telelactation users (n=31), n (%)	*P* value
**Maternal sociodemographics**				
	Marital status (married)	33 (52)	17 (55)	.82
	Race (Caucasian)	60 (95)	30 (97)	.73
	High school degree or less	31 (49)	13 (42)	.51
	Smartphone ownership at baseline (yes)	61 (97)	30 (97)	.99
	Private insurance during pregnancy	29 (46)	16 (52)	.62
	Public insurance during pregnancy	31 (49)	14 (45)	.72
**Childbirth and breastfeeding characteristics and plans**				
	Plan to work in baby’s first year (yes)	29 (46)	20 (65)	.09
	Working by 12 weeks	26 (41)	21 (68)	.02
	First time mom (yes)	22 (35)	17 (55)	.07
	Caesarean section delivery	28 (44)	16 (52)	.52
	Baby sex (boy)	39 (62)	17 (55)	.52
	Baby delivered > 37 weeks	54 (86)	27 (87)	.86
	Prior breastfeeding experience (yes)	41 (65)	12 (39)	.02
	Plans to breastfeed exclusively for 12+ weeks	54 (86)	26 (84)	.66
	Breastfed exclusively while in hospital	51 (81)	16 (52)	<.001
**Maternal health and risk factors**				
	Smoking at 12 weeks	12 (19)	6 (19)	.97
	Depression (prepregnancy)	15 (24)	6 (19)	.63
	Anxiety (prepregnancy)	21 (33)	6 (19)	.16
	Obesity (prepregnancy)	5 (8)	4 (13)	.45
	Hypertension (prepregnancy)	7 (11)	1 (3)	.20

Compared to participants who had one or more telelactation video calls that included discussion of a breastfeeding challenge, those who did not engage in calls were similar on most sociodemographic, maternal health, childbirth, and breastfeeding characteristics. However, participants who used telelactation were more likely to be working at 12 weeks postpartum (21/31, 68%; *P*=.02) compared to mothers who did not use telelactation (26/63, 41%) and less likely to have prior breastfeeding experience (12/31, 39%; *P*=.02) on average compared to nonusers (41/63, 65%). They were also less likely to have breastfed exclusively prior to discharge from the hospital (16/31, 52%; *P*<.001) relative to mothers who did not use telelactation (51/63, 81%), as assessed at the baseline survey during the postpartum hospitalization.

### Characteristics of Video Calls

According to vendor EMR data, 31 participants in the telelactation arm completed a total of 83 video calls that addressed one or more breastfeeding challenges. Characteristics of these video calls are presented in Table 2. The average video call duration was 7 minutes and 19 seconds (SD 5.5 mins). Most calls (58/83, 70%) occurred during the infant’s first month of life; however, 19% (16/83) of video calls took place nine or more weeks after the infant’s birth. In total, 41% (34/83) of video calls occurred outside of regular business hours (defined as any time between 8 AM and 6 PM, excluding weekends), and 59% (49/83) addressed more than one breastfeeding challenge or concern. The most common challenges discussed on video calls included: breast pain, soreness, and infection (25/83, 30% of calls), use of nipple shields (21/83, 25%), latch or positioning (17/83, 24%), milk supply and production (14/83, 17%), and the use of a breast pump (14/83, 17%).

**Table 2:** Characteristics of video calls.

Characteristic		Value, n (%)
**Age of infant when call occurred**		
	<7 days	37 (45)
	2-4 Weeks	21 (25)
	5-8 Weeks	11 (13)
	9-12 Weeks	6 (7)
	13+ Weeks	10 (12)
**Time of day of call**		
	8 AM-12 PM	23 (28)
	12 PM-4 PM	29 (35)
	4 PM-8 PM	22 (27)
	8 PM-8 AM	9 (11)
	During business hours (yes)	49 (59)
**Topics discussed on calls**		
	Breast pain, soreness, infection	25 (30)
	Use of nipple shields	21 (25)
	Latch or positioning	17 (24)
	Milk supply and production	14 (17)
	Breast pump use	14 (17)
	Infant condition or health	13 (16)
	Infant weight or whether getting enough milk	10 (12)
	Mother’s return to work	9 (11)
	Mother’s illness or medications interacting with breastfeeding	8 (10)
	Mother’s dietary restrictions	7 (8)
	Infant uninterested in eating	4 (5)

### Experiences With and Attitudes About Telelactation Among Self-Reported Telelactation Users

According to survey data from the final 12-week assessment, 47/94 (50%) of participants who were given access to telelactation did not participate in any video calls. Leading reasons for not participating in any video calls for breastfeeding support during the study included: not experiencing any breastfeeding problems (26% of nonusers), not being comfortable with video calls (11%), no longer breastfeeding or stopped breastfeeding during the study (8%) and being too busy (7%).

At the 12-week assessment, the majority of telelactation users indicated that the breastfeeding assistance they received via the app was helpful (41/47, 87%) and expressed satisfaction with the help received (43/47, 91%). Of the 47 telelactation users, 9 (19%) had one or more recommendations for improving the app. Of these participants, four suggested that the app should allow mothers to text with IBCLCs and two users requested an option to do audio only calls. Each of the following recommendations was offered by one user, who suggested that the app should allow users: 1) to request a particular IBCLC; 2) to connect to breastfeeding peers; 3) to access blogs and other written resources on breastfeeding; or 4) to automatically schedule monthly calls that the mother does not need to initiate.

Over the course of the study, 10 participants reported attempting to use the app without success. We included these participants along with self-reported telelactation users (n=57) to identify any technical difficulties encountered in requesting or participating in video calls. A total of three participants (7%) reported difficulties in finding or connecting to wireless internet. No other technical problems (eg, dropped calls, poor sound quality, long wait for IBCLCs) were identified by more than one participant. Of all the participants, 36/47 (78%) did not report experiencing any technical difficulties.

## Discussion

This study revealed relatively robust demand for telelactation, particularly in the first weeks after delivery, and user satisfaction with telelactation services. Video calls tended to be much shorter in duration (7 minutes on average) than a scheduled, in-person visit with an IBCLC. Also, video calls often took place outside of business hours, suggesting that participants were taking advantage of the scheduling flexibility supported by this delivery model. Telelactation users were relatively similar to nonusers; however, users were less likely to be breastfeeding exclusively prior to discharge from the hospital and also less likely to have prior breastfeeding experience. These findings suggest that telelactation services may be particularly attractive to mothers who lack the confidence and skills that come with prior breastfeeding experience as well as those experiencing early difficulties at the time of initiation.

Forty-five percent of participants with self-reported breastfeeding challenges and 31% of all eligible participants requested and participated in substantive telelactation visits that addressed breastfeeding issues. In addition, 50% reported engaging with the app in some manner. This rate of uptake is dramatically higher than observed for other telehealth interventions offered to a population, and for telephone-based breastfeeding support (uptake rate of 0.5-24%) [25-28]. We attribute the relatively high demand for telelactation in the context of this study to several factors. First, according to baseline survey data on breastfeeding plans, the population of mothers that participated in the trial were committed to long duration, exclusive breastfeeding, and relatedly, to overcoming any challenges that arose. This was the case in part because women who did not intend to breastfeed following hospital discharge were excluded from the trial. At the time of enrollment, 84% (26/31) of telelactation arm participants indicated that they planned to breastfeed exclusively for 12 or more weeks, as compared to 57% of pregnant women nationally who reported plans to breastfeed exclusively for the first few weeks [29]. Second, telelactation services were introduced to potential users by trusted healthcare providers (ie, nurses caring for participants in person during the postpartum hospitalization), and participants were given an orientation to the app at the time of enrollment that often included a test call.

While we could not identify any research that describes the content of telelactation visits via personal devices, numerous studies both within and outside of the United States have assessed telephone breastfeeding support visits [30-35]. The top three issues discussed in video calls in this study included: breast pain, soreness, or infection, use of nipple shields, and latch or positioning. No prior analyses of telephone visits identified nipple shield use as a common topic, however, main reasons for visits seemed to vary depending on the patient population. For example, common issues discussed by 908 callers to a helpline in the United Kingdom included difficulties with positioning and concerns about inadequate milk supply [30]. In contrast, common issues identified in an analysis of 1969 calls to an inner-city hospital breastfeeding support line in the United States included questions about obtaining and using a breast pump, and breast issues [31]. Numerous questions about nipple shields in this study may reflect the local practices of healthcare professionals at the recruitment hospital during the postpartum hospitalization.

This work builds upon our prior qualitative work that showed that telelactation is acceptable and feasible for rural mothers. While select participants in both analyses recommended the addition of audio only and text message visit options, one participant in the current analysis also indicated a desire for regularly scheduled video calls that the breastfeeding mother did not have to initiate. At this point, this form of telelactation requires the mother to identify and seek help for a breastfeeding issue. Prior research has differentiated between proactive (prescheduled at regular intervals) and reactive (as demanded by the mother) breastfeeding support and suggested that proactive support can lead to greater engagement and impact [36]. Future telelactation models can consider how best to deliver services that are responsive to urgent needs but also require less activation from mothers who could benefit from professional advice.

The primary limitation of this study is that we recruited participants at one study site that serves a population of rural, predominantly Caucasian mothers in Pennsylvania. As such, it is unclear how patterns of telelactation use may differ in other communities with different breastfeeding support services. Nonetheless, the study was conducted in the context of a randomized controlled trial and is the first of its kind to use quantitative methods to explore use of and experiences with telelactation.

Telelactation is an innovation in the delivery of professional breastfeeding support. Although our study focused on a rural population, these services may increase convenience and reduce costs associated with seeking in-person breastfeeding support in urban settings as well. Although additional research should document the impacts of these services on breastfeeding outcomes and healthcare costs within more diverse populations, this research documents demand for and positive experiences with telelactation in an underserved population.

## Abbreviations

EMR: electronic medical record
HIPAA: Health Insurance Portability and Accountability Act
HPSA: Health Professional Shortage Area
HRSA: Health Resources and Services Administration
IBCLC: International Board-certified Lactation Consultant
IRB: Institutional Review Board
